# At what cost? The impact of bacteriophage resistance on the growth kinetics and protein synthesis of *Escherichia coli*

**DOI:** 10.1111/1758-2229.70046

**Published:** 2024-11-19

**Authors:** Lotta A. I. Landor, Jesslyn Tjendra, Karen Erstad, Anders K. Krabberød, Joachim P. Töpper, Selina Våge

**Affiliations:** ^1^ Department of Biological Sciences University of Bergen Bergen Norway; ^2^ Marine Biological Section, Department of Biology University of Copenhagen Helsingør Denmark; ^3^ Department of Biosciences University of Oslo Oslo Norway; ^4^ Norwegian Institute for Nature Research Bergen Norway

## Abstract

Cost of bacteriophage resistance (COR) is important in explaining processes of diversification and coexistence in microbial communities. COR can be expressed in different traits, and the lack of universally applicable methods to measure fitness trade‐offs makes COR challenging to study. Due to its fundamental role in growth, we explored protein synthesis as a target for quantifying COR. In this study, the growth kinetics of three genome‐sequenced strains of phage‐resistant *Escherichia coli*, along with the phage‐susceptible wild‐type, were characterized over a range of glucose concentrations. Bioorthogonal non‐canonical amino acid tagging (BONCAT) was used to track differences in protein synthetic activity between the wild‐type and phage‐resistant *E. coli*. Two of the resistant strains, with different levels of phage susceptibility, showed mucoid phenotypes corresponding with mutations in genes associated with the Rcs phosphorelay. These mucoid isolates, however, had reduced growth rates and potentially lower protein synthetic activity. Another resistant isolate with a different mutational profile maintained the same growth rate as the wild‐type and showed increased BONCAT fluorescence, but its yield was lower. Together, these findings present different patterns of trade‐offs resulting from the phage‐induced mutations and demonstrate the potential applicability of BONCAT as a tool for measuring COR.

## Graphical abstract

Bacteriophage resistance in *Escherichia coli* can come at the cost of decreased or even increased bacterial growth rate, yield, and protein synthetic activity as quantified through BONCAT. This study presents different patterns of fitness trade‐offs resulting from various phage‐induced mutational profiles and demonstrates the potential applicability of BONCAT as a tool for measuring the cost of resistance.

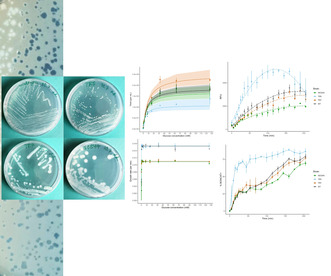

## INTRODUCTION

In microbial communities, interaction dynamics between bacterial host cells and lytic bacteriophage (phage), an example of top‐down control, is a major driver of diversity (Brockhurst et al., 2005; Sandaa et al., 2009; Storesund et al., 2015; Weinbauer & Rassoulzadegan, 2004), along with bottom‐up control through resource specialization (Dal Bello et al., 2021; Ekkers et al., 2022; Sun & Sanchez, 2023). Theoretical studies predict evolutionary diversification as being strongly influenced by the nature of trade‐offs between bottom‐up and top‐down factors (Thingstad, 2000; Våge et al., 2013). For instance, the form of trade‐offs between competitive and defensive abilities in bacterial hosts has been shown to influence long‐term coexistence and potential for diversification in host–phage communities (Pourhasanzade et al., 2022). In marine environments, the abundance of phage and thus, partitioning of bacterial production between transport to higher trophic levels and recycling within the microbial loop, is predicted to depend on the trade‐off between competition for limiting resources and defence against lytic phage (herein referred to as cost of resistance, COR; Brockhurst et al., 2005; Record et al., 2016). COR thus plays a role in the biogeochemical functions of the marine microbial food web (Våge et al., 2016, 2018).

Despite strong theoretical predictions for COR to be important for various aspects of the microbial food‐web organization, experimental evidence for it remains sparse and inconclusive (Litchman & Klausmeier, 2008). One challenge is that the expression of trade‐offs depends on environmental conditions (Bohannan et al., 2002). Another challenge is that methods to quantify trade‐offs in environmental settings are under‐developed. Also, trade‐offs may appear in different traits or phenotypes (Litchman et al., 2013), which warrants a description of resistance effects beyond classical growth rate measurements.

Protein synthesis is a central process for bacterial growth, as cells tightly regulate their protein economy to provide building blocks for the growing cells. Changes in protein synthetic activity could thus be an attractive target for the determination of fitness implications in phage‐resistant bacteria. A promising method for this purpose is bioorthogonal non‐canonical amino acid tagging (BONCAT), which uses amino acid analogues that can be selectively labelled using a copper(I)‐catalysed azide‐alkyne cycloaddition reaction (Dieterich et al., 2006; Rostovtsev et al., 2002). The method can be used to detect translational activity in single cells without the need for culturing, which allows application in studies of protein synthetic activity in diverse microbial communities (Couradeau et al., 2019; Hatzenpichler et al., 2014, 2016; Leizeaga et al., 2017; Lindivat et al., 2020; Reichart et al., 2020; Samo et al., 2014). However, as BONCAT has not previously been used for the purpose of quantifying COR, the method needs to be assessed using a simpler model system before it can be applied to complex microbial communities.

This study aims to evaluate the applicability of BONCAT in quantifying COR by tracking protein synthetic activity in phage‐resistant cultures of *Escherichia coli* under laboratory conditions. For comparison with traditional approaches, we characterised growth parameters (maximum growth rate, maximum yield, and nutrient affinity) of the phage‐resistant isolates, and analysed their genotypes through whole genome sequencing. We demonstrate that different genotypes amongst the resistant strains manifested into different phenotypic signatures, highlighting the importance of studying the costs of resistance using methods that can differentiate COR profiles, especially in mixed populations. We also suggest a receptor site for phage G28.

## EXPERIMENTAL PROCEDURES

### Media

Minimal M9 media were prepared from autoclave sterilized M9 salts with the working concentrations of 48 mM Na_2_HPO_4_·2H_2_O, 22 mM KH_2_PO_4_, 28 mM NH_4_Cl, and 8.6 mM NaCl. To the salts, autoclave sterilized CaCl_2_ and MgSO_4_·8H_2_O solutions were added to final concentrations of 0.1 and 2 mM, respectively. A 2 M glucose stock was prepared in RO‐H_2_O and sterile filtered. M9 media was prepared fresh before each experiment and the glucose concentration was adjusted accordingly. Agar plates were prepared from M9 media with 11 mM glucose and 1.5% agar. Soft agar was prepared from M9 media, 11 mM glucose, and 0.5% agar and decanted into 4 mL aliquots.

### Bacterial isolates, storage, and culture conditions

Wild‐type *E. coli* DSM 103246 (hereby called WT) was obtained from the German Collection for Microorganisms and Cell Cultures (DSMZ). The strain is of serotype O186:H34 and was originally isolated for a phage therapy study in chickens (Kittler et al., 2020; Korf et al., 2020; Schmidt et al., 2017). Glycerol stocks of the strain were produced from a single colony inoculated in M9 medium (5.6 mM glucose) and incubated at 37°C overnight. Following incubation, four parts culture was mixed with one part glycerol and frozen in 500 μL aliquots at −80°C. For each experiment, liquid culture was prepared by inoculating M9 media with a thawed glycerol stock, followed by incubation at 37°C overnight.

Escherichia phage vB_EcoM‐G28 (DSM 103876, hereby called G28) was obtained from DSMZ as a liquid suspension. The phage is lytic and classified as a *Tequatrovirus* (Korf et al., 2020). A “starter lysate” was prepared by adding the phage suspension into an exponentially growing host culture (WT), incubated under 150 rpm agitation at 37°C in M9 media (5.6 mM glucose). Following complete lysis, the lysate was collected by centrifugation (5445 × *g*, 3 min) and sterile filtered (0.45 μm). The lysate was stored at 4°C in the dark.

### Single‐plaque lysate preparation

Lysate originating from a single plaque was prepared using an adapted agar method described by the DSMZ (Leibniz‐Institut DSMZ, 2023), which consistently yielded lysate in the range of 10^9^ PFU/mL for phage G28. First, a dilution series was performed on the starter lysate in glucose‐free M9. Then, an equal volume of overnight culture of the susceptible WT host (optical density, 600 nm; OD_600_ = 0.2) and a dilution of the G28 starter lysate were mixed. Immediately after mixing, 300 μL of the phage–host mix was mixed in 4 mL molten (50°C) soft M9 agar and poured over room temperature M9 agar plates. The plates were incubated overnight at 37°C. Following incubation, plates showing clear and separate plaques were chosen and single plaques were isolated using the tip of a sterile pipette. The single plaque was suspended in 200 μL glucose‐free M9.

A double‐layer agar plate of the susceptible host was prepared by mixing 300 μL of overnight culture (adjusted to OD_600_ = 0.2) of WT in 4 mL molten (50°C) soft M9 agar and poured over an M9 agar plate. The single‐plaque suspension was then transferred and spread over the surface of the double‐layer agar plate and incubated overnight at 37°C. Following incubation, phages were harvested from the resulting lysed area in the bacterial lawn by pipetting 10 mL of glucose‐free M9 onto the plate and slowly rotating the plate at room temperature for 5–6 h. The liquid on the plate was then transferred into a sterile tube and centrifuged for 3 min at 5445 × *g*. The supernatant was sterile filtered (0.45 μm), and the resulting lysate was stored at 4°C.

### Isolation of phage‐resistant mutants

Phage G28‐resistant mutants were isolated through a double‐layer agar method, whereby phage‐susceptible WT strain exposed to high concentration of the phage in soft agar was poured over an M9 agar plate. Briefly, an overnight culture of WT was adjusted to OD_600_ = 0.2 and mixed with an equal volume of G28 lysate (10^7^–10^9^ PFU/mL). Immediately after mixing, 300 μL of the phage–host mix was pipetted into 4 mL molten (50°C) soft M9 agar, vortexed, and poured over an M9 agar plate. After the soft agar had solidified, the plate was incubated at 37°C overnight. The following day, single colonies growing on top of the soft agar were isolated and streaked onto a phage‐free M9 agar plate. The isolates were passaged through further isolation of single colonies 2–3 times on agar to clean them from contaminant phage. The phage susceptibility of all isolated colonies was tested using spot assays. Out of all the isolates, 4–5 were found to have changed susceptibility to the phage after a single exposure effort to roughly 10^6^–10^8^ PFU of the phage (300 μL of the phage–host mixture).

### Efficiency of plating

Efficiency of plating (EOP), as a measure of phage susceptibility of the isolated strain, was determined by calculating the ratio of the average plaque forming units (PFU) of the G28 lysate formed on the isolated strain to the average PFU formed on the wild‐type strain (Liao et al., 2019). The average PFU of the lysate was determined through plaque assays, as described in the following. A dilution series was performed on the lysate in glucose‐free M9 media. An overnight culture (M9 media with 5.6 mM glucose, 37°C) of the test bacterium was then adjusted to OD_600_ = 0.2, added into each dilution of the lysate in the series at an equal volume, and vortexed gently. 300 μL of each phage–host mixture was pipetted into 4 mL molten (50°C) soft M9 agar, vortexed, and poured over an M9 agar plate. The plates were incubated at 37°C overnight, and PFU formed in the lawn of test bacterium was counted for every plate with consistent appearance of separate, countable plaques. Every plaque assay was performed with a wild‐type control, and with 3–5 replicate plates per dilution of the lysate.

Phage susceptibility was classified based on guideline by Hancock and Reeves (1975) for complete (EOP < 10^−7^) and partial (EOP ≥ 10^−2^) phage resistance, and an intermediate category for distinctively lower susceptibility level than the threshold for partial resistance.

### Spot assay

A qualitative double‐layer agar spot assay was used to confirm phage susceptibility of isolated strains before and after experiments. Unless mentioned otherwise, 300 μL of overnight culture (M9 media with 5.6 mM glucose, 37°C) of the test bacterium (adjusted to OD_600_ = 0.2) was mixed into 4 mL of molten (50°C) soft M9 agar and poured over an M9 agar plate. A dilution series was performed on the G28 lysate in glucose‐free M9 media. Each dilution was pipetted into three replicate 5 μL drops on the surface of the soft agar top layer. Each spot assay was done as three replicate plates per test bacterium. In spot assays measuring phage susceptibility, the test bacterium (isolated strain) was tested in parallel with a wild‐type control. The plates were incubated overnight at 37°C. Following incubation, the plaques at the terminal dilution, determined through the consistent appearance of separate, countable plaques, were counted.

### Optimal carbon stoichiometric estimation and glucose concentrations

Glucose concentrations for the assays were chosen based on the stoichiometric relationship between carbon (C), nitrogen (N), and phosphate (P) in M9 media. Standard M9 salts contained an N:P ratio of 19:70. Based on a simple stoichiometric model (Fagerbakke et al., 1996; Scott et al., 2012), we estimated the optimal C:N:P ratio to be 100:10:1. Under this optimal ratio, M9 media would become N‐limited above a C‐concentration of 190:19 C:N, whereas P would remain in excess. Glucose as a carbon source contains six C per molecule. Therefore, the optimal glucose concentration for bacterial cultures in M9 media was calculated to be 31.7 mM glucose, equivalent to 190:19 C:N.

### Microwell plate glucose assay

Cultures of *E. coli* WT and G28‐resistant mutants were grown in M9 media containing 5.6 mM glucose, and incubated overnight at 37°C. Following incubation, the cultures were centrifuged at 5445 × *g* for 5 min and the supernatant, containing any trace glucose, was removed. The cultures were resuspended in glucose‐free M9 and adjusted to OD_600_ = 0.2. The OD_600_‐adjusted cultures were diluted 1:100 in glucose‐free M9 media. The phage susceptibility of all strains was tested from the OD_600_‐adjusted cultures using spot assays.

To the wells of a 96‐well flat‐bottomed microwell plate, 20 μL of the diluted cultures were added to 180 μL M9 media containing a gradient of glucose concentrations. Four rows of the wells were added with the WT strain, and the remaining four rows were added with a resistant strain. The final glucose concentrations after culture addition per column of wells were 127, 63, 32 (optimal), 16, 8, 4, 2, 1, 0.5, and 0.25 mM. The two highest concentrations of glucose were N‐limited, whereas concentrations below 32 mM glucose were C‐limited. In addition, a glucose‐free control (0 mM) and a bacteria‐free control (containing 14 mM glucose) were included on the plate.

The plate was placed in a pre‐warmed (37°C) 2300 EnSpire™ Multilabel Reader (PerkinElmer). The plate reader was set to read the OD_600_ of the culture in every well for 18 h with 20 min intervals and at 37°C. Immediately after the last read, the contents of the microwell plate were sampled for flow cytometric cell count and spot assay. End‐of‐assay spot assays were performed on pooled wells for each bacterial strain. The assay was repeated three times for each WT and resistant strain pairing, resulting in nine replicates for WT strain and three replicates for each resistant strain.

### Flow cytometric count

Final yields of *E. coli* from the glucose assays were determined through flow cytometric counts from two pooled wells for each glucose treatment and each bacterial strain. The samples were fixed using 25 μL/mL 37% formaldehyde solution (252549, Sigma‐Aldrich). The fixed samples were diluted in sterile‐filtered (0.2 μm) Tris–EDTA (TE) buffer and stained with 10 μL/mL SYBR green I (Thermo Fisher Scientific) for 10 min in the dark. The samples were analysed using the Attune NxT Flow Cytometer (Thermo Fisher Scientific) with a blue laser 488 nm (50 mV). The detector BL1 was used to detect the SYBR‐fluorescence at excitation and emission wavelength at 530 and 30 nm, respectively, and at a flow rate of 200 μL/min. The number of cells was determined through gating of SYBR‐positive events.

### Growth kinetics analysis

All data were processed and analysed using Excel version 2308, Python version 3.6.5 (Van Rossum & Drake, 2009), or R version 4.3.1 (R Core Team, 2023). The growth rate was determined by calculating the slope of the linear regression through four or five consecutive optical density (OD_600_) data points, with basis on the following formula: $\mu =\left(\ln N_{t}-\ln N_{0}\right)/\left(t-t_{0}\right)$ whereby *μ* is the growth rate (min^−1^); *N*_*t*_ is the OD_600_ at time *t*; *N*_0_ is the OD_600_ at time *t*_0_; *t* is the time (min); *t*_0_ is the initial time (min). The OD data points were selected through visual assessment of the onset of exponential growth in the OD curve, roughly 400–500 min into the assay.

In obtaining estimates of the nutrient affinity and maximum growth rate of *E. coli*, the statistical methods non‐linear least squares (R‐package “minpack.lm”; Elzhov et al., 2023) and generalized least squares (R‐package “nlme”; Pinheiro et al., 2023) were used to fit the following equation onto the growth rate data $y=\mathrm{ax}/\left(1+\left(\mathrm{ax}/b\right)\right)$ whereby *y* is the growth rate (*μ*; /min^−1^), *x* is the glucose concentration (mM), *α* is the nutrient affinity (min/mM), *b* is the maximum growth rate (*μ*_max_; min^−1^; asymptote). The equation was obtained by substituting *K*_*s*_ for $V_{\max}/\alpha$ in the Monod model (Kovárová‐Kovar & Egli, 1998; Monod, 1949). The fitted non‐linear regression was built using generalized least squares with a modified variance structure (model argument ‘VarIdent’; different variance per glucose concentration) due to heteroscedasticity of the growth rate data across glucose concentration.

The same equation was fitted onto the flow cytometric count data to provide an estimation of the maximum yield, whereby *y* is the final yield (mL^−1^), *x* is the glucose concentration (mM), and *b* is the maximum yield (mL^−1^; asymptote). Generalized least squares was again used to fit the non‐linear regression (additional R‐package “nlraa” for bootstraping; Miguez, 2023), with the variance structure modified (model argument ‘VarConstPower’; constant plus power of the variance covariate) to correct for heteroscedasticity of the final yield data across glucose concentration.

For comparison of growth rate or final yield amongst the strains at a particular glucose concentration, one‐way ANOVA and the post‐hoc analysis Tukey HSD were run provided that data was homoscedastic across strains.

### BONCAT experiment set up: time course incubation

Overnight cultures of the wild‐type and phage‐resistant mutants were prepared in M9 media (5.6 mM glucose) at 37°C. The cultures were then adjusted to OD_600_ of 0.2. Spot assays were performed for the wild‐type and resistant strains to confirm phage susceptibility. From the OD_600_‐adjusted cultures, 2 mL was added to 198 mL of pre‐warmed M9 (37°C; 5.6 mM glucose). To the culture flasks (one wild‐type and one phage‐resistant strain per experiment run), L‐azidohomoalanine (AHA; 1066, Click Chemistry Tools) was added to a final concentration of 50 μM. This concentration was chosen due to satisfactory fluorescence and negligible effect on bacterial growth (Landor et al., 2023). The flasks were incubated at 37°C.

Upon AHA‐addition, the flasks were immediately sampled and the 1 mL samples (in triplicates from each flask, plus one 1‐mL cell count sample at each sampling point) were fixed with 25 μL of 37% formaldehyde solution for cell counting. The flasks were subsequently sampled at appropriate intervals for a total of 3.5 h. The samples collected for cell counting were flash‐frozen in liquid nitrogen without further processing. The other samples were centrifuged at 16,000 × *g* for 5 min, and the supernatant containing excess AHA and fixative was removed. To avoid disproportionate loss of BONCAT‐negative cells, cells were resuspended in sterile phosphate‐buffered saline (PBS, pH = 7.4) containing 20% glycerol as cryoprotectant before flash freezing in liquid nitrogen. This sample processing was done within 30 min of each sampling time‐point. All samples were transferred to −20°C until further processing and analysis. After the last sampling point, spot assays were performed on the remaining cultures to confirm phage susceptibility. A total of 85 mL was sampled from each flask during the experiment.

The experiment was run three times for each WT and resistant strain pairing to give a total of nine replicates for WT strain and three replicates for each resistant strain. As it had previously been verified that fixed (killed) cells of *E. coli* DSM 103246 do not produce BONCAT‐fluorescent cells (data not shown), killed controls were not included in this experiment set‐up.

### BONCAT‐staining

The staining protocol published by Landor et al. (2023) was used with minor adjustments. The frozen samples were allowed to thaw at room temperature in the dark. Glycerol was removed from the sample by centrifugation at 16,000 × *g* for 10 min and the supernatant was replaced with sterile‐filtered PBS. To each 1 mL sample, 57 μL each of freshly made water solutions of 100 mM sodium ascorbate (A4034, Merck) and 100 mM aminoguanidine hydrochloride (396494, Sigma‐Aldrich) were added. A dye premix was prepared and incubated for 3 min in the dark, before adding to each sample, giving the final concentrations of 120 μM CuSO_4_ (C8027, Sigma‐Aldrich), 570 μM THPTA (1010, Click Chemistry Tools) and 5.7 μM AlexaFluor™ 647 alkyne dye (1301, Click Chemistry Tools). The samples were mixed avoiding aeration and incubated for 1 h in the dark. Following incubation, the samples were centrifuged for 5 min at 16,000 × *g* and the supernatant was replaced with TE buffer containing 0.02% Tween‐20. The samples were stored at 4°C in the dark until flow cytometric analysis (within 24 h of staining).

### Flow cytometric analysis of BONCAT samples (BONCAT‐FCM)

BONCAT‐stained cells were further stained with SYBR Green I (Thermo Fisher Scientific), briefly vortexed, and incubated for 10 min in the dark. Each sample was analysed three times with an Attune NxT Flow Cytometer (Thermo Fisher Scientific), using both a blue (488 nm, 50 mV) and red laser (638 nm, 50 mV) at dye‐specific excitation/emission wavelengths (SYBR 530/30 nm, AlexaFluor 670/14 nm) and at 100 μL/min flow rate. Bacterial cells were identified through gating of SYBR‐positive events, from which the BONCAT‐positive fraction of the population was distinguished using quadrant gating (adapted from Lindivat et al., 2020). The fluorescence threshold for BONCAT‐positive events (in relative fluorescence units, RFU) was determined using the negative control as reference. The fraction of BONCAT‐active cells (%) was calculated by dividing the number of BONCAT‐positive events by the total number of SYBR‐positive events.

Cell count samples from each sampling time‐point were thawed at room temperature and transferred to 4°C on the day of flow cytometric analysis. Each sample was diluted 1:10 and 1:100 in sterile‐filtered (0.2 μm) TE buffer and stained with SYBR Green I (Thermo Fisher Scientific) for 10 min in the dark before being analysed using a blue laser 488 nm (50 mV) on the Attune NxT Flow Cytometer. SYBR‐fluorescence at excitation and emission wavelengths of 530 and 30 nm, respectively, was detected with BL1. The samples were analysed at the same flow rate as the BONCAT samples (100 μL/min).

Data collected with the flow cytometer were analysed using Excel version 2308 and R version 4.3.1 (R Core Team, 2023). A mean value of the background fluorescence (i.e., negative control; in RFU) was subtracted from all corresponding data. A quadratic linear mixed effects regression (R‐packages “lme4”; Bates et al., 2015) with Gaussian errors and identity link was then fitted onto the RFU data. In the model, strain and incubation time were specified as fixed effects, whereas random intercepts and slopes were estimated for the sampled cultures. Additional R‐package “multcomp” (Hothorn et al., 2008) was used for post‐hoc Tukey HSD analysis of the model. All other data comparisons at particular time‐points were done using one‐way ANOVA and Tukey HSD.

### Whole genome sequencing and analysis

Samples for DNA extractions were obtained by centrifugation (14,000 × *g*, 5 min) of 2–5 mL of overnight culture grown in M9 media (5.6 mM glucose) and resuspended in sterile‐filtered (0.2 μm) PBS. Spot assays were performed on all cultures before extraction to confirm phage susceptibility. The DNA extractions were done using Wizard® Genomic DNA Purification Kit (A1120, Promega) following the manufacturer's instructions for Gram‐negative bacteria (Technical Manual TM050, 2019). The extracted DNA samples were adjusted to 30.6 ± 3.7 ng/μL DNA for quality estimation using NanoDrop One (Thermo Fisher Scientific) and Qubit 2.0 High Sensitivity dsDNA kit (Thermo Fisher Scientific). The average 260/280 and 230/260 ratios for the extracted DNA were 1.89 and 2.00, respectively. The DNA samples were visualized on a 0.75% agarose gel stained with GelRed® Nucleic Acid Gel Stain (41003, Biotium), run for 5 h at 60 V.

Library preparation and sequencing were conducted by the Norwegian Sequencing Centre. DNA was sheared into 10–15 kb fragments using g‐tubes (Covaris). Libraries were constructed using the SMRTbell® ExpressTemplate Prep Kit 2.0, following the Pacific Biosciences protocol for Multiplexed Microbial Libraries (PN 101‐742‐600 version 4, 2021). Fragments without the SRMTbell adapters were removed post‐ligation through an additional nuclease treatment. Samples were pooled together in equimolar ratio.

The entire library was sequenced on an 8M SMRT cell on the Sequel IIe System with Sequel II Binding Kit 2.2 and Sequencing Chemistry v2.0. Resulting HiFi reads were demultiplexed to the specific samples, generating a mean of 1.1 × 10^9^ bases per strain with an average read length of 8307 ± 2885 bp (Appendix S2).

Genome analysis was performed via Geneious Prime® 2023.1.2 (Dotmatics). Raw HiFi reads were mapped to a reference sequence (accession no. CP019944) using the alignment algorithm Minimap2 (Li, 2018).

## RESULTS

### Efficiency of plating and colony morphology

Three phage G28‐resistant mutant strains, designated TR5, TR7, and ISO24A, were isolated and used in this study (Table 1). The mutant TR5 had similar colony morphology on agar compared to the wild‐type (WT; Figure S1). In liquid cultures, TR5 grew suspended in the media but notably produced more sediment than WT. The mutants TR7 and ISO24A, on the other hand, appeared as mucoid colonies, but no change to sedimentation was noted.

**TABLE 1:** The average efficiency of plating (EOP) of wild‐type and phage G28‐resistant *E. coli* strains.

Strain	EOP
DSM 103246 (WT)	1.0
TR5	1.8 × 10^−6^
TR7	3.9 × 10^−2^
ISO24A	2.5 × 10^−8^

### Mutations

Different mutations were found in the different phage‐resistant strains (Table 2; Appendix S2). In isolate TR5, an insertion of approximately 1199 base long was predicted to result in truncation of O‐antigen polymerase (Wzy, accession no. AQZ76922.1). TR5 also had an additional insertion of approx. 1338 base long in another gene, predicted to cause truncation of the protein disulfide isomerase I (AQZ80000.1), also known as thiol:disulfide oxidoreductase. A single nucleotide polymorphism (SNP) in TR7 led to the amino acid substitution of lysine (K) into asparagine (N) within the receiver or REC domain (codons 828–941) of a hybrid sensory kinase (RcsC, AQZ76732.1). ISO24A also had an SNP that led to amino acid substitution (glycine → glutamic acid) within a homologue of the membrane protein IgaA (YrfF, AQZ75446.1). No change was detected within the CRISPR arrays of any of the resistant strains to indicate activation of the defence system in response to phage infection. Note that the wild‐type and phage‐resistant strains carried a few additional mutations from the GenBank‐deposited reference sequence (CP019944.1; Appendix S2).

**TABLE 2:** Mutations from the wild‐type (WT) in the phage G28‐resistant *E. coli* strains.

Strain	Mutation	Locus	Variant	Coding sequence
TR5	Insertion	1,977,235	1199 base long insert	*wzy* (O‐antigen polymerase)
TR5	Insertion	5,283,895	1338 base long insert	*dsbA* (disulfide isomerase I/thiol:disulfide oxidoreductase)
TR7	SNP (transversion)	1,761,229	G → C (amino acid K → N)	*rcsC* (hybrid sensory kinase)
ISO24A	SNP (transition)	416,646	C → T (amino acid G → E)	*yrfF* (homologue of the membrane protein IgaA)

### Growth kinetics and phenotypes

**FIGURE 1:**
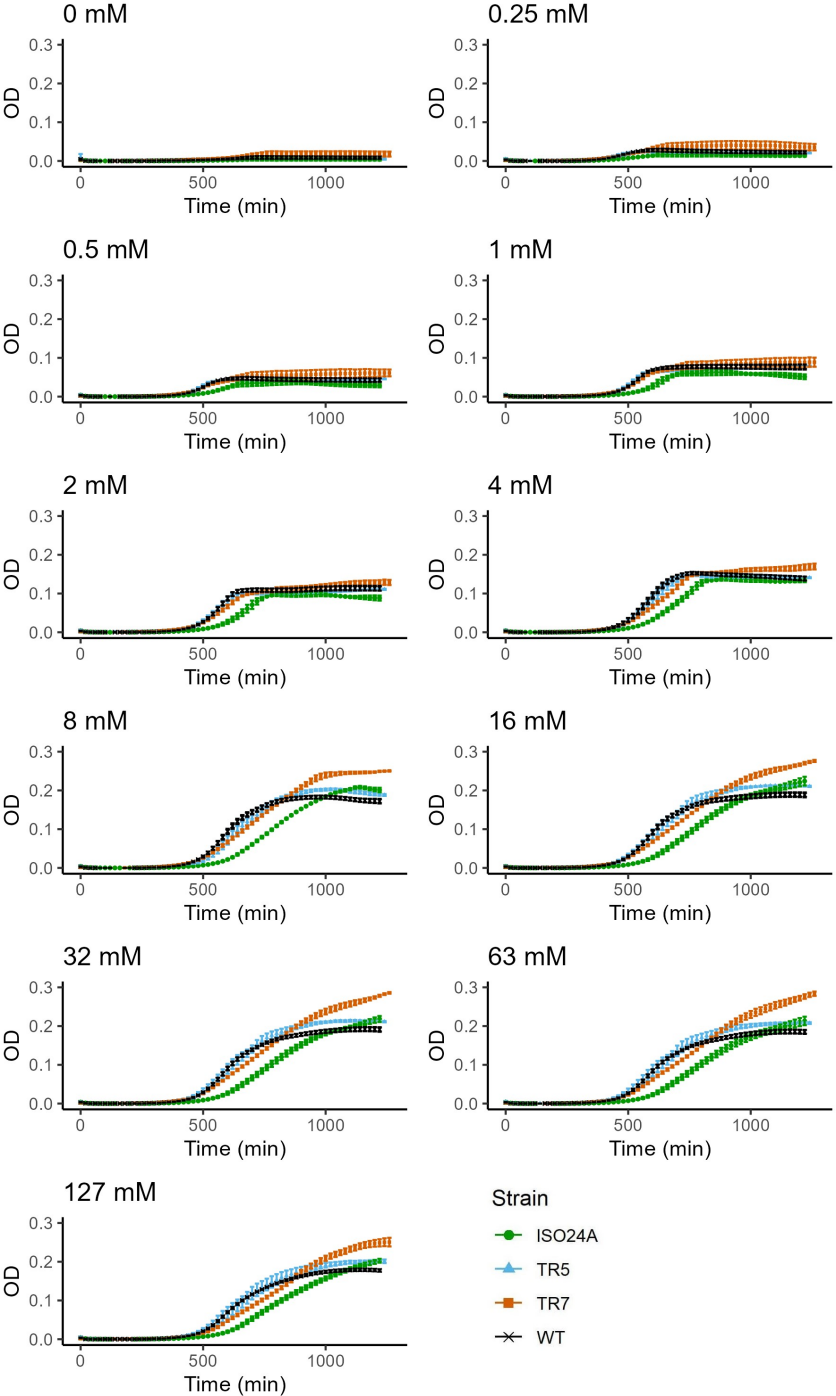
Growth curves of *E. coli* DSM 103246 wild‐type (WT) and phage G28‐resistant strains TR5, TR7 and ISO24A assayed at varying glucose concentrations (0–127 mM) in M9 media. Growth was defined as an increase in culture turbidity, measured through its optical density (OD) at 600 nm. Datapoints = mean (*n* = 3; *n*_WT_ = 9), error bars = ±1 SEM.

Glucose supported *E. coli* growth for all strains whilst the absence of glucose resulted in inconsistent and minimal growth across the assays, with culture turbidity (OD_600_) increase of only up to ca. 0.03 (Figure 1). Maximum (i.e., stationary phase) turbidity reached by the culture correlated positively with glucose concentration in the media up to 8 mM, after which turbidity remained similar regardless of the concentration. TR7 was consistently observed to have the highest turbidity amongst the strains as the cultures reached stationary growth. ISO24A displayed the slowest increase in turbidity before it eventually caught up to WT and TR5 at stationary phase and reached similar turbidity within the duration of the assays.

**FIGURE 2:**
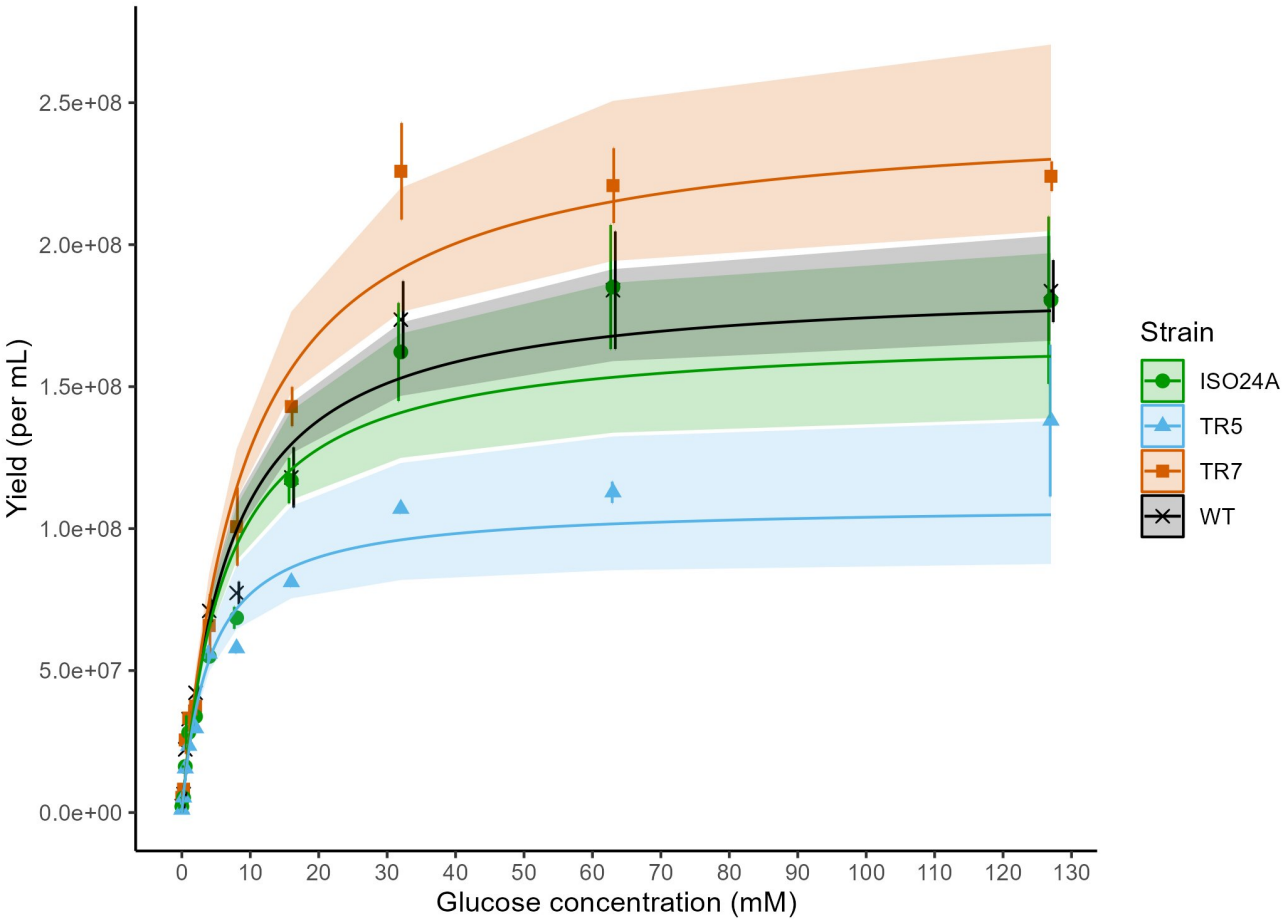
Final yield, as flow cytometric cell count per mL culture, of *E. coli* DSM 103246 wild‐type (WT) and phage G28‐resistant strains TR5, TR7, and ISO24A grown at a range of glucose concentrations (0–127 mM) in M9 media. Datapoints = mean (*n* = 3; *n*_WT_ = 9), error bars = ±1 SEM, lines = fitted non‐linear regression, bands = 90% quantile confidence around fitted regression.

The yield data from flow cytometric total counts of *E. coli* cells at the end of assay revealed comparable pattern of relative abundances (Figure 2). At higher glucose concentrations (≥8 mM), TR7 produced the most cells amongst the strains. TR5, on the other hand, had the least even though its maximum turbidity was higher compared to that of WT. Based on a non‐linear regression fitted using generalized least squares, the maximum yields that could be reached with glucose in excess were estimated to be 2.47, 1.86, 1.69, and 1.08 × 10^8^ cells/mL culture for strains TR7, WT, ISO24A, and TR5, respectively. These estimates differed significantly (*p* < 0.05) from one another except between WT and ISO24A (*p* = 0.2532). Using the same fitted regression, the yields at 5.6 mM glucose corresponding to the concentration in the BONCAT experiments were estimated to be 9.30, 8.29, 7.92, and 6.27 × 10^7^ cells/mL for strains TR7, WT, ISO24A, and TR5, respectively.

**FIGURE 3:**
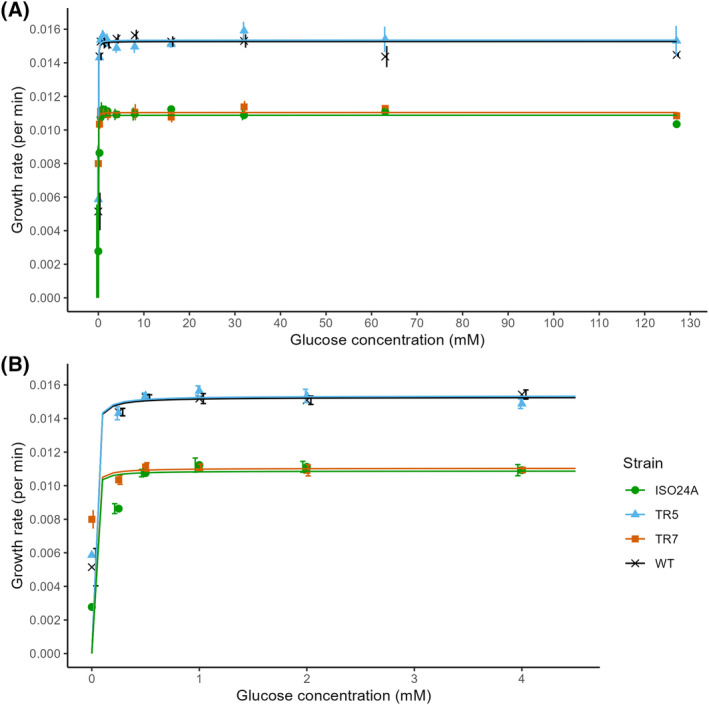
Growth rates (min^−1^) of wild‐type *E. coli* DSM 103246 (WT) and phage G28‐resistant *E. coli* strains TR5, TR7, and ISO24A cultured in (A) 0–127 mM glucose. (B) Magnification of the chart for 0–4 mM glucose. Datapoints = mean (*n* = 3; *n*_WT_ = 9), error bars = ±1 SEM, lines = fitted regression.

The growth rate was significantly lower for TR7 and ISO24A than for WT and TR5 at 0.5 mM glucose and above (Tukey HSD, *p* < 0.05; Figure 3). TR5 did not differ from the wild‐type in its growth rate at any of the glucose concentrations tested (Tukey HSD; *p* > 0.05). The maximum growth rates (*μ*_max_) were 1.54, 1.53, 1.10, and 1.09 × 10^−2^ min^−1^ for TR5, WT, TR7, and ISO24A, respectively, as estimated through a generalized least squares regression fitted on the data. The strains were observed to have reached their maximum growth rates already by 0.5 mM glucose. Estimates of the nutrient affinity based on a non‐linear least squares model decreased from 1.86 (min mM)^−1^ for WT to 1.15, 0.84, and 0.22 (min mM)^−1^ for TR5, TR7, and ISO24A, respectively. However, these estimates were not statistically significant.

Phage susceptibility/resistance of the strains remained unchanged throughout the glucose assays, as confirmed through spot assays of the cultures before and after the glucose assays.

### BONCAT

**FIGURE 4:**
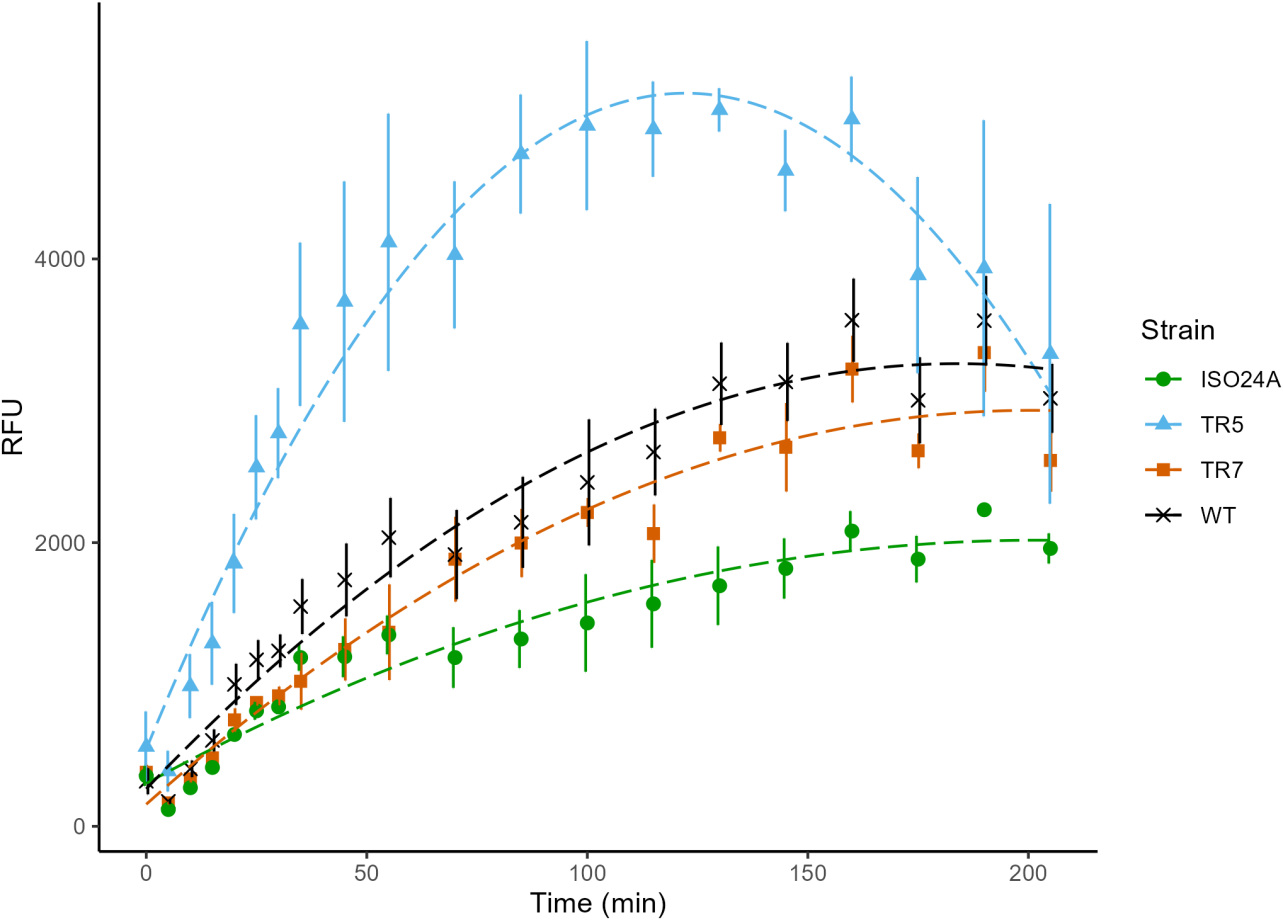
BONCAT fluorescence per cell in relative fluorescence units (RFU) of wild‐type *E. coli* DSM 103246 (WT) and phage G28‐resistant *E. coli* strains TR5, TR7 and ISO24A incubated with 50 μM L‐azidohomoalanine (AHA). Datapoints = mean (*n* = 3; *n*_WT_ = 9), error bars = ±1 SEM, dashed line = fitted regression.

In general, BONCAT signal per cell, defined in terms of relative fluorescence unit (RFU), increased over time for all strains, before levelling off, or even decreased (TR5), towards the end of the incubation period (205 min; Figure 4). According to a quadratic linear mixed effects model fitted onto the data, only TR5 was found to differ significantly from the other strains in its BONCAT fluorescence (Tukey HSD, *p* < 0.05). There were, however, a couple time‐points (130 and 160 min) during which strain ISO24A showed significantly lower fluorescence per cell than WT (Tukey HSD, *p* = 0.04 and *p* = 0.03, respectively). TR5, on the other hand, emitted significantly higher fluorescence per cell, except at the start (0 min) and from 175 min onwards.

**FIGURE 5:**
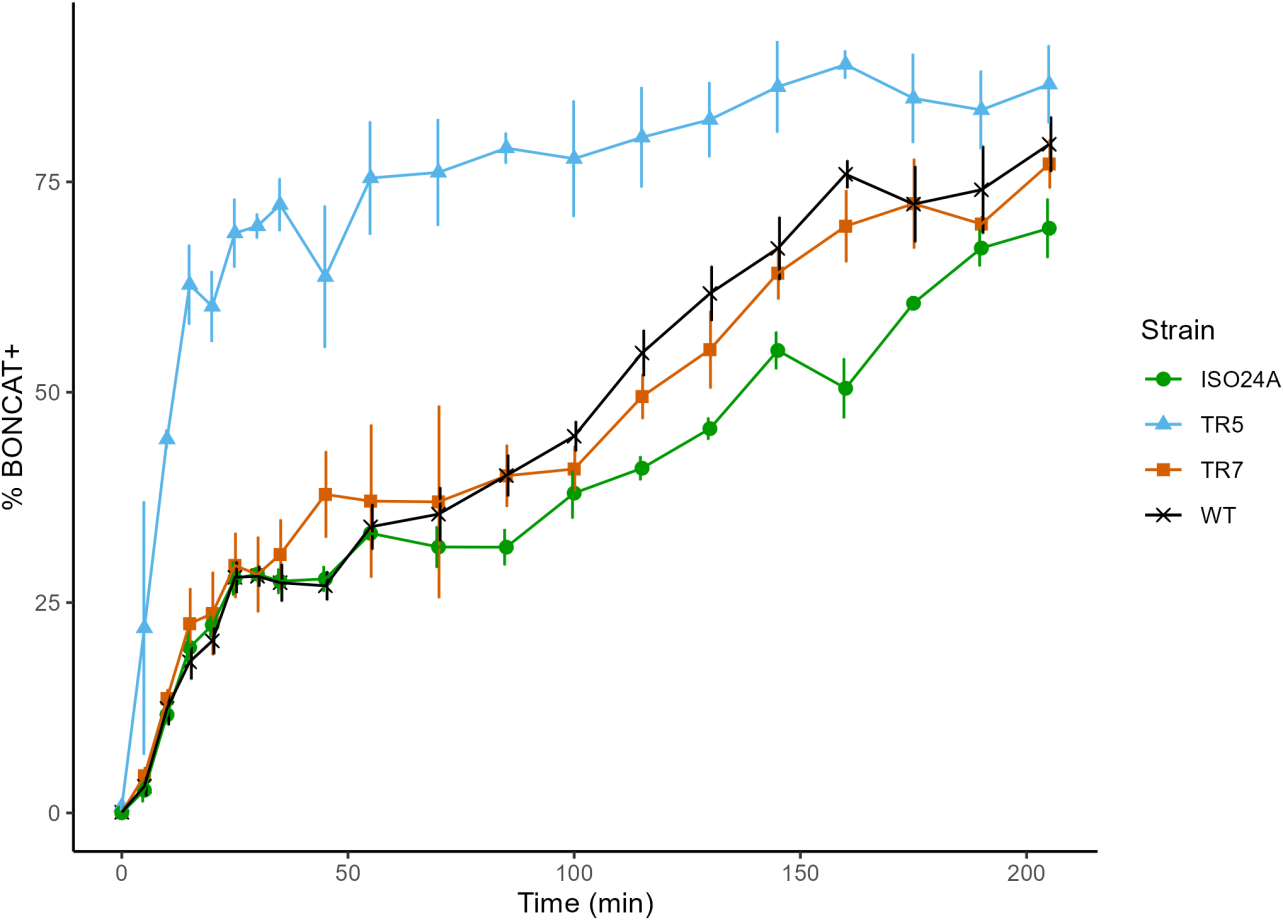
Active fraction (%) of the wild‐type *E. coli* DSM 103246 (WT) and phage G28‐resistant (TR5, TR7 and ISO24A) *E. coli* populations, based on cells emitting BONCAT fluorescence (BONCAT+), over the duration of incubation in 50 μM L‐azidohomoalanine (AHA). Datapoints = mean (*n* = 3; *n*_WT_ = 9), error bars = ±1 SEM.

The emergence of BONCAT‐positive cells started immediately after AHA addition. All strains followed a similar pattern of a rapid increase in the fraction of active cells at the start of the incubation, which then stalled for a period after ca. 30 min, before increasing again through to the end of incubation (Figure 5). The early leap was most pronounced for TR5, which reached 70% BONCAT‐positive cells 30 mins into the incubation and remained the highest (Tukey HSD, *p* < 0.05) up until 160 min. No significant difference was found between TR7 and the wild‐type at any point during the incubation. ISO24A, on the other hand, had the lowest (Tukey HSD, *p* < 0.05) percentage of active cells amongst the strains for a brief period (160–175 min) during the incubation.

## DISCUSSION

As the bulk mass of bacterial cells consists of proteins (Stouthamer, 1973), bacterial growth strongly correlates with protein synthesis, which could account for more than 60% of the ATP budget during the exponential growth phase (Bosdriesz et al., 2015; Klumpp et al., 2013; Russell & Cook, 1995). The resistant strain TR5 showed distinctively higher BONCAT signal per cell (Figure 4), indicating higher translational activity (Hatzenpichler et al., 2014; Leizeaga et al., 2017), as well as proportion of active cells (Figure 5). The high BONCAT fluorescence, and thus energetic cost of protein production, could hypothetically exert trade‐offs in TR5, potentially manifesting in the lower maximum yield of the isolate (Figure 2). We suspect that the mutations that rendered the strain phage‐resistant also caused the cells to increase protein synthetic activity and maintain their growth rate (compared to WT; Figure 3) at the cost of a lower output of abundance (i.e., inefficiency in biomass production; Beardmore et al., 2023). The growth assays were designed such that 32 mM glucose provided a theoretically ideal stoichiometry of carbon and nitrogen, and an increase in protein synthetic activity may have initiated nitrogen limitation prior to 32 mM glucose.

One possible explanation for the high BONCAT fluorescence of TR5 cells is the insertion causing a truncation of disulfide isomerase I (thiol:disulfide oxidoreductase, DsbA), an enzyme involved in the catalysis of disulfide bonds essential for correct folding of proteins (Bardwell et al., 1991; Hiniker et al., 2005; Inaba et al., 2006). *E. coli* has been hypothesized to increase expression and availability of Dsb‐enzymes to accommodate for the protein needs of growing cells (Rettenbacher & von der Haar, 2022). Whilst methionine (AHA being a methionine analogue) does not form disulfide bonds, it is the most frequent amino acid for protein synthesis initiation in *E. coli* (Kozak, 1983). Cells might therefore compensate for the debilitating effects of the mutation on DsbA activity by upregulating the initiation of protein synthesis, as reflected in the increased uptake of the methionine analogue and, thus, BONCAT fluorescence.

The fully resistant strain ISO24A periodically had the least BONCAT fluorescence and the lowest proportion of active cells, whereas the partially resistant strain TR7 showed slightly (albeit insignificantly) lower fluorescence compared to WT (Figures 4 and 5). Both strains showed distinct mucoid phenotypes (Figure S1), and mucoidy is known to be capable of providing general resistance against phage by blocking physical contact with the cell surface (Chaudhry et al., 2020). As a mode of defence, mucoidy is often associated with partial resistance and spontaneous reversion due to the cost of overproduction of the exopolysaccharides—thus implicating cost of resistance to heterogeneity in bacterial populations and coevolution with phage (Harrison et al., 2015; Scanlan & Buckling, 2012; Wielgoss et al., 2016). Consistent with our results for TR7 and ISO24A (Table 3), mucoidy has previously been associated with reduced growth rate and, hence, relative fitness of cells (Scanlan & Buckling, 2012). The lower growth rates and, potentially, protein synthetic activities of these two mucoid strains compared to WT could indicate a more energetically efficient means to maximize growth and produce comparable or even higher yields under existing constraints. As an example of how this rate‐yield trade‐off could be mediated, several studies on glucose metabolism have shown that reduced nutrient uptake decreases the rate, but increases the efficiency, of adenosine triphosphate (ATP) production (Beardmore et al., 2023; Meyer et al., 2015; Novak et al., 2006). In this capacity, BONCAT alone may not be sufficient to illuminate such a trade‐off in rate‐yield relationship in ISO24A or TR7.

**TABLE 3:** An overview of trait differences (+ increase; − decrease; = no change; parentheses () indicate statistical non‐significance) of the phage G28‐resistant strains compared to the wild‐type *E. coli* DSM 103246.

	TR5	TR7	ISO24A
Phage resistance^a^	Intermediate	Partial	Complete
Colony morphology	Smooth	Mucoid	Mucoid
Maximum growth rate	=	−	−
Maximum yield	−	+	=
Nutrient affinity	(62%)	(45%)	(12%)
BONCAT fluorescence per cell	+	(−)	(−)
BONCAT active cells	+	=	−

*Note*: Nutrient affinity is expressed in percentage compared to the wild‐type (100%).

^a^ Phage resistance was based on efficiency of plating (EOP) values, using Hancock and Reeves (1975) classification as a guideline to set partial (EOP ≥ 10^−2^) and complete (EOP < 10^−7^) resistance.

The inhibition of phage adsorption through modification or blocking of cell surface receptors might have implications for the labelling efficiency of phage‐resistant bacteria. The BONCAT staining reagents are of small molecular size that allow penetration through cell walls without the need for permeabilization steps (Samo et al., 2014), but permeabilization treatments have been shown to increase labelling efficiency (Leizeaga et al., 2017). The mutation impacting the O‐antigen polymerase (Wzy) could have implications for labelling efficiency of TR5 by reducing its mechanical O‐antigen barrier (Delcour, 2009; Kulikov et al., 2017). Similarly, phage resistance through mucoidy could hinder the BONCAT reagents from readily penetrating through the extracellular matrix. It is therefore possible that the significantly higher BONCAT fluorescence in TR5, and the generally lower BONCAT fluorescence of ISO24A and TR7, could be the results of a differential labelling efficiency rather than increased/reduced protein synthetic rates. Given that surface modification is a common mode of phage resistance with potentially variable impact on cell surface permeability, the use of permeabilization steps (e.g., Leizeaga et al., 2017) might be a necessary addition to the BONCAT protocol for the quantification of COR.

BONCAT has shown great promise in tracking protein synthetic activity in environmental communities (Hatzenpichler et al., 2020), and its application in studying virus–host interactions has only begun to be realized. Adding the quantification of COR to the versatile range of BONCAT applications, for example in measuring viral production, tracking amino acid transfer between host and virus (Pasulka et al., 2018), and in detecting phage–host complexes (Hellwig et al., 2024), can broaden the understanding of bacterium‐phage dynamics in natural ecosystems. The application of BONCAT to quantify COR in environmental communities beyond the laboratory remains to be tested. This study showed that phage G28 can select for several different genotypic outcomes with corresponding unique phenotypic profiles in *E. coli*. In a natural population of *E. coli* cells being exposed to phage G28, it is therefore likely that multiple phage‐resistant genotypes would coexist. If COR can be expressed as both increased and reduced BONCAT signal in individual genotypes (as observed in this study), the ability of BONCAT to detect the COR of individual genotypes would rely on the fluorometric instrument's ability to separate the different cell populations based on their unique BONCAT signals. For this purpose, fluorescence‐based cell sorting (BONCAT‐FACS; Hatzenpichler et al., 2016) can be a promising approach for quantification of COR in bacterial communities.

### The Rcs phosphorelay appears important for phage G28 interaction with its host

The *E. coli* DSM 103246 and phage G28 model system was relatively recently introduced to science (Kittler et al., 2020; Korf et al., 2020) and the virus–host interactions of the system are not yet understood. Whilst it is not an aim of this study to fully elucidate the molecular mechanisms for phage adsorption or resistance, our genomic analysis hints at a potentially shared mechanism mediating resistance to phage G28.

In two of the G28‐resistant isolates, ISO24A and TR7, the mutations were found in genes directly associated with the Rcs (*r*egulation of *c*apsular polysaccharide *s*ynthesis) phosphorelay pathway shared by many members of *Enterobacterales* (Wall et al., 2018). A point mutation was found in ISO24A that ought to cause amino acid substitution (i.e., missense mutation) within YrfF, a homologue of the inner membrane protein IgaA, an essential switch for the Rcs signalling circuit that has regulatory functions, particularly in biofilm formation, motility, and virulence (Wall et al., 2018, 2020). An *igaA* (*yrfF*) mutant with mucoid phenotype has indeed been previously observed in *E. coli*, also associated with upregulation of several components of the Rcs phosphorelay as well as genes in the colanic acid biosynthesis operon (Mutalik et al., 2020). The same study demonstrated that the mutation in *igaA* conferred resistance at varying levels to a myriad of phages, which was attributed to overproduction of the colanic acid polysaccharide limiting accessibility of the membrane receptor for phage adsorption (Chaudhry et al., 2020; Hancock & Reeves, 1975; Scholl et al., 2005). Based on these findings, we postulate that the mucoidy of ISO24A is a result of high expression of colanic acid due to the point mutation in the IgaA homologue.

The partially resistant mutant TR7 carried a missense mutation in the hybrid sensor kinase RcsC, another core component of the IgaA‐regulated Rcs phosphorelay. Mutations in *rcsC* are known to bring about constitutive Rcs activity, presumably due to interactions with other auxiliary components in the pathway (Pannen et al., 2016; Wall et al., 2018). Majdalani et al. (2005) linked a missense mutation (*rcsC137*) to elevated expression of the capsule synthesis operon *cps* and a highly mucoid phenotype, suggesting a potential mechanism for how the point mutation in TR7 led to its mucoidy. However, the different levels of phage G28 resistance observed for TR7 and ISO24A (Table 1) provide some indication that the mechanism of resistance may extend beyond the blockage of the receptor due to capsular polysaccharides.

Assuming that TR7 and ISO24A had similar mucoidy, the difference in their level of phage resistance indicates that there may be other factors implicated in the resistance aside from a capsular polysaccharide barrier. The mutations in TR5 could potentially confer a more precise phage resistance mechanism. However, TR5 differs from the other mutants not only by the nature of the mutational changes (>1 kb inserts), but also in that the strain showed mutations at two distinct loci (Table 2). We speculate that the insertion observed in *dsbA* could interfere with the Rcs system by impacting disulfide bond formation in RcsF, the outermost component of the pathway responsible for environmental sensing (Kadokura et al., 2004; Wall et al., 2018). All of the G28‐resistant isolates thus share a feature of similarity in that they all carried mutations that potentially impacted Rcs activity.

Seemingly unrelated to the Rcs phosphorelay, TR5 also showed an insertion within *wzy*, which was predicted to lead to truncation of the O‐antigen polymerase. The O‐antigen is not essential for *E. coli* growth under laboratory conditions, and the laboratory strain *E. coli* K‐12 lacks the O‐antigen (Liu & Reeves, 1994; Stevenson et al., 1994). *E. coli* strains lacking the O‐antigen are known to have a distinct “rough” colony morphology (Lerouge & Vanderleyden, 2002; Stenutz et al., 2006), and *E. coli wzy*‐mutant Nissle 1917 (DSM 6601) has been shown to display “semi‐rough” (i.e., smooth and rough) colonies (Grozdanov et al., 2002). Surprisingly, the colonies of TR5 appeared smooth and undistinguishable from the wild‐type (Figure S1). A previous study on *E. coli* harbouring a deletion within the *wzy* gene (mutant DE17Δ*wzy*) showed that the phenotype of the LPS as well as the serum agglutination of the O‐antigen were affected (Zuo et al., 2019). Based on these findings, we hypothesize that TR5 may have altered O‐antigen configuration or lack the O‐antigen completely.

It should be noted that the O‐antigen is generally considered as a barrier for phage‐infection (Letarov, 2023; Maffei et al., 2021). Whilst some *E. coli*‐infecting phages (identified to e.g., *Drexlerviridae* and T5‐like phages) use the O‐antigen as a receptor (Golomidova et al., 2016; Maffei et al., 2021), no phages belonging to *Tequartoviridae* have been shown, to the best of our knowledge, to use the O‐antigen for host recognition. In fact, restoration of the O‐antigen in *E. coli* K‐12 is associated with wide‐ranging phage resistance, specifically against phages belonging to *Tequartoviridae* (Maffei et al., 2021). Moreover, phage G28 is not dependent on the O‐antigen for adsorption, as it is known to be capable of infecting *E. coli* K‐12 lacking the O‐antigen (Korf et al., 2020). It is possible that the insertion in *wzy* in TR5 occurred independently from the G28‐interaction, possibly during the cleaning steps after G28‐exposure, and might be a result of adaptation to laboratory conditions, similar to what has been hypothesized for the O‐antigen loss in the laboratory strain K‐12 (Liu & Reeves, 1994). It is also possible, that the *wzy*‐mutation is a compensatory mutation to reduce wide‐ranging impacts of the mutation in *dsbA* (Hiniker & Bardwell, 2004; Hiniker et al., 2005; Lee et al., 2008; Yamanaka et al., 1994), although the compensatory mechanism is unclear.

Apart from the isolates in this study, another G28‐resistant strain has previously been characterised (Korf et al., 2020). This mutant showed a deletion affecting proteins proposed to be important for phage adsorption: a MotB domain protein of the outer membrane porin A (OmpA) family and a putative outer membrane protein of the OmpC family. Whilst both OmpA and OmpC are known receptors to *Tequartoviridae* phages (Maffei et al., 2021), OmpA, rather than OmpC, is known to form a complex with RcsF, thereby preventing RcsF from interacting with IgaA and activating the phosphorelay (Cho et al., 2014; Dekoninck et al., 2020). Furthermore, OmpA is a substrate of DsbA (Santos‐Martin et al., 2021). The putative truncation of DsbA in TR5 could hypothetically affect the folding and hence structure of OmpA, preventing efficient phage recognition and/or adsorption. We therefore hypothesize that OmpA is the receptor for phage G28. However, given the fact that all three G28‐resistant mutants showed unique genotypic profiles, it is likely that phage G28 infection is a multifactorial process.

Finally, whole‐genome sequencing revealed that our wild‐type *E. coli*, as well as all three phage‐resistant mutants, had five additional mutations from the *E. coli* DSM 103246 reference sequence submitted by Schmidt et al. (2017; accession no. CP019944.1). Consequently, it is important to keep in mind that these additional mutations could potentially result in physiological changes that differentiate our wild‐type strain from *E. coli* DSM 103246 analysed in other studies.

## CONCLUSION

This study demonstrates that *Escherichia coli* resistance to phage G28 can manifest as differences in BONCAT fluorescence, suggesting that the method may be applicable for quantifying the cost of resistance (COR). The study also shows that phage resistance can come at the cost of decreased (or even increased) bacterial growth rate and yield, both of which would impact the host's ability to compete for resources. However, further investigation is needed to validate whether BONCAT fluorescence accurately reflects proteomic differences in the phage‐resistant *E. coli*. We found that a common resistance mechanism appears to be linked to mutations affecting core components of the Rcs phosphorelay and upregulation of capsular polysaccharide synthesis. Resistance may also be conferred through an unknown mechanism associated with the O‐antigen and/or periplasmic protein folding machinery, possibly involving the outer membrane porin A (OmpA) as a candidate receptor for phage adsorption. The different patterns of fitness trade‐offs in our resistant isolates highlight that methods aimed at measuring COR need to be able to distinguish between different genotypes in a mixed population, which BONCAT might have the capacity to do.

## AUTHOR CONTRIBUTIONS

**Lotta A. I. Landor:** Conceptualization; investigation; writing – original draft; methodology; validation; visualization; writing – review and editing; formal analysis; project administration; data curation; supervision; resources. **Jesslyn Tjendra:** Conceptualization; investigation; writing – original draft; methodology; validation; visualization; writing – review and editing; software; formal analysis; project administration; data curation; resources. **Karen Erstad:** Investigation; methodology; writing – review and editing; data curation. **Anders K. Krabberød:** Methodology; investigation; writing – review and editing; software; formal analysis; data curation; conceptualization; validation; supervision; resources. **Joachim P. Töpper:** Conceptualization; methodology; validation; writing – review and editing; supervision; resources. **Selina Våge:** Conceptualization; investigation; funding acquisition; writing – original draft; methodology; validation; writing – review and editing; project administration; supervision; resources.

## CONFLICT OF INTEREST STATEMENT

The authors declare no conflicts of interest.

## Supporting information


**FIGURE S1:** Colony morphology of wild‐type *Escherichia coli* DSM 103246 (A), compared to phage G28‐resistant isolates TR5 (B), TR7 (C), and ISO24A (D) on M9 agar after 32 h incubation at 37°C. TR5 shows a similar morphology to the wild‐type, whilst TR7 and ISO24A show a mucoid colony morphology.
**FIGURE S2:** Plaques of phage G28 in the lawn of wild‐type *Escherichia coli* DSM 103246 (A, B), phage G28‐resistant isolates TR5 (C, D), TR7 (E), and ISO24A (F) on M9 agar after approx. 24 h incubation at 37°C. Plaque(s) formed on TR5 and ISO24A appeared considerably smaller and fainter in comparison to those formed on WT.
**FIGURE S3:** Flow cytometric count (per mL) of the wild‐type *E. coli* DSM 103246 (WT) and phage G28‐resistant (TR5, TR7 and ISO24A) *E. coli* cells over the duration of incubation in 50 μM L‐azidohomoalanine (AHA). Datapoints = mean (*n* = 3; *n*_WT_ = 9), error bars = ±1 SEM.

**APPENDIX S2:** Supplementary information.

## Data Availability

The genomic data from this study has been deposited into the European Nucleotide Archive (ENA) under study accession no. PRJEB73472. All other relevant data are available through the repository Open Science Framework (OSF): https://osf.io/9c7t3/.
