# Supplementary material for: At what cost? The impact of bacteriophage resistance on the growth kinetics and protein synthesis of Escherichia coli
Source: Environ Microbiol Rep. 2024 Nov 19;16(6):e70046. doi: 10.1111/1758-2229.70046 (PMC11576411; doi:10.1111/1758-2229.70046)
Supplement: Supplementary file 1 — FIGURE S1: Colony morphology of wild‐type Escherichia coli DSM 103246 (A), compared to phage G28‐resistant isolates TR5 (B), TR7 (C), and ISO24A (D) on M9 agar after 32 h incubation at 37°C. TR5 shows a similar morphology to the wild‐type, whilst TR7 and ISO24A show a mucoid colony morphology. FIGURE S2: Plaques of phage G28 in the lawn of wild‐type Escherichia coli DSM 103246 (A, B), phage G28‐resistant isolates TR5 (C, D), TR7 (E), and ISO24A (F) on M9 agar after approx. 24 h incubation at 37°C. Plaque(s) formed on TR5 and ISO24A appeared considerably smaller and fainter in comparison to those formed on WT. FIGURE S3: Flow cytometric count (per mL) of the wild‐type E. coli DSM 103246 (WT) and phage G28‐resistant (TR5, TR7 and ISO24A) E. coli cells over the duration of incubation in 50 μM L‐azidohomoalanine (AHA). Datapoints = mean (n = 3; n WT = 9), error bars = ±1 SEM. [file EMI4-16-e70046-s001.docx]

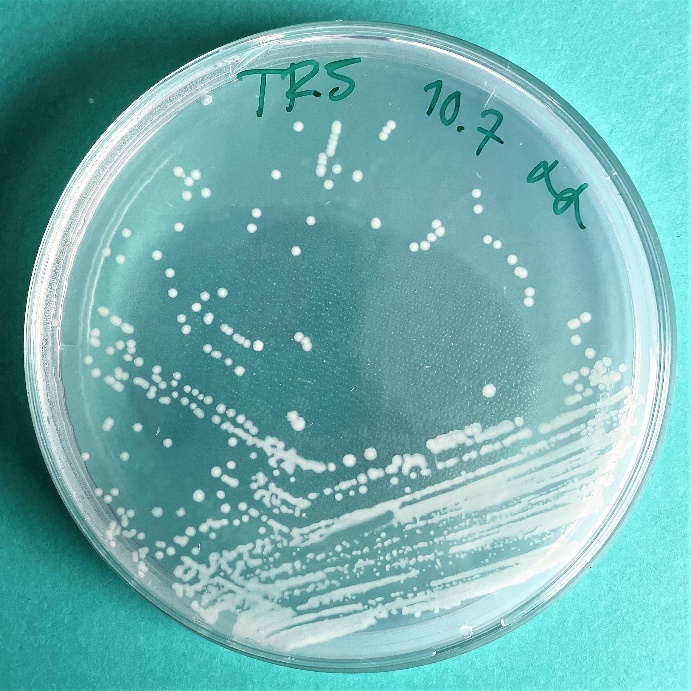

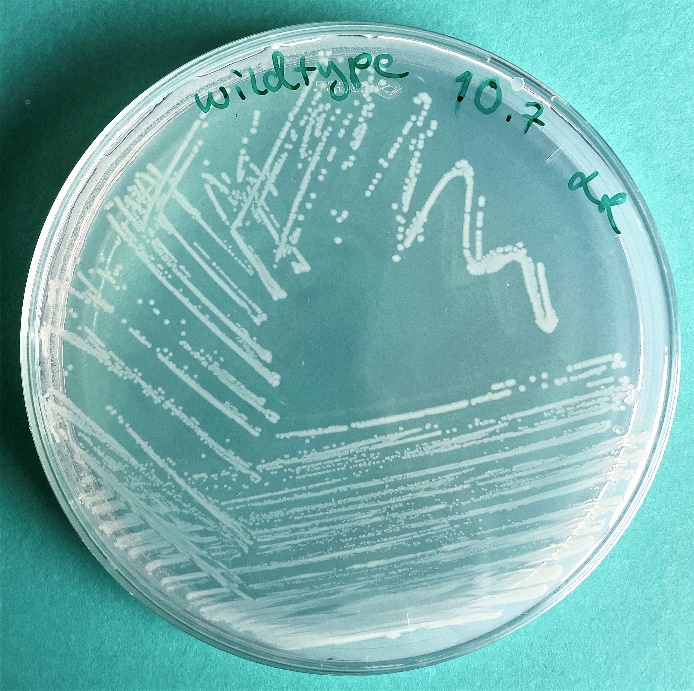

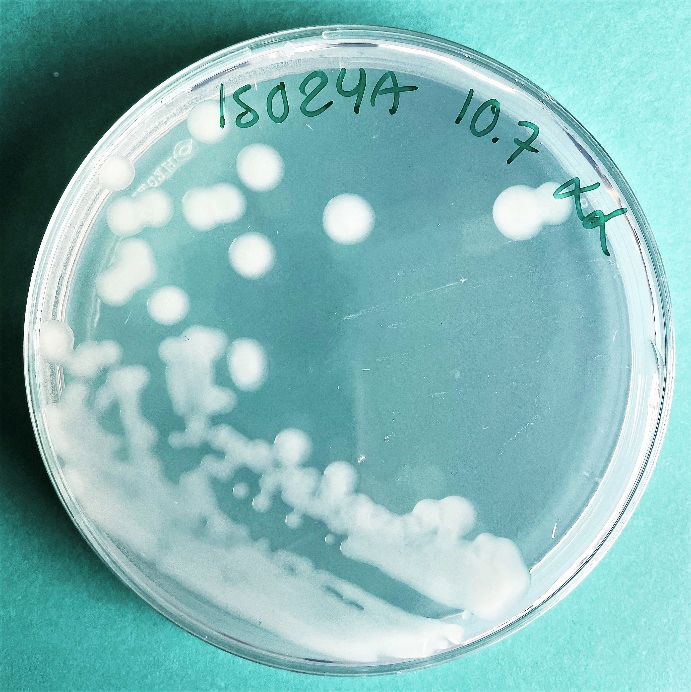

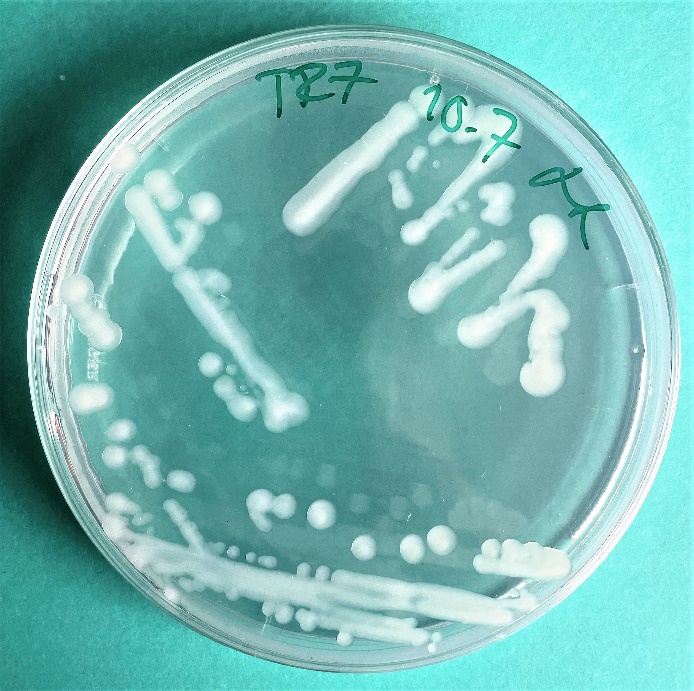


A B

C D

**Figure S1.** Colony morphology of wild-type *Escherichia coli* DSM 103246 (A), compared to phage G28-resistant isolates TR5 (B), TR7 (C), and ISO24A (D) on M9 agar after 32 h incubation at 37 °C. TR5 shows a similar morphology to the wild-type, while TR7 and ISO24A show a mucoid colony morphology.


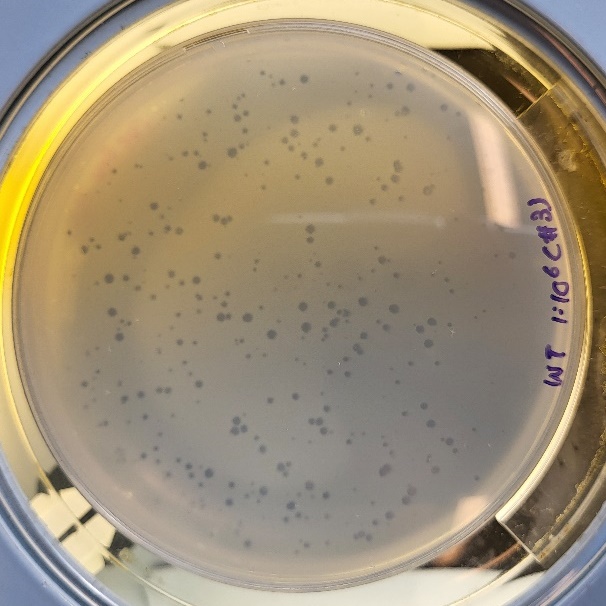

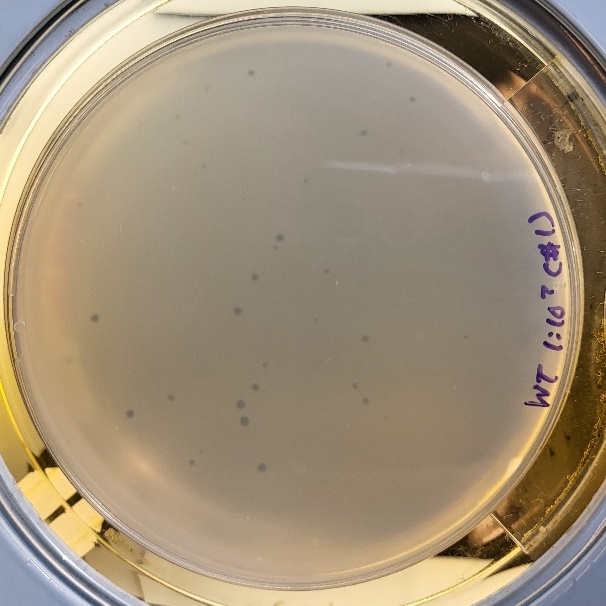

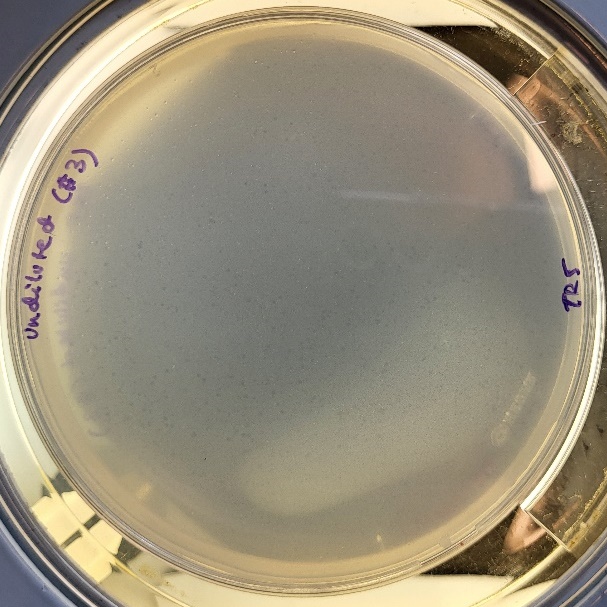

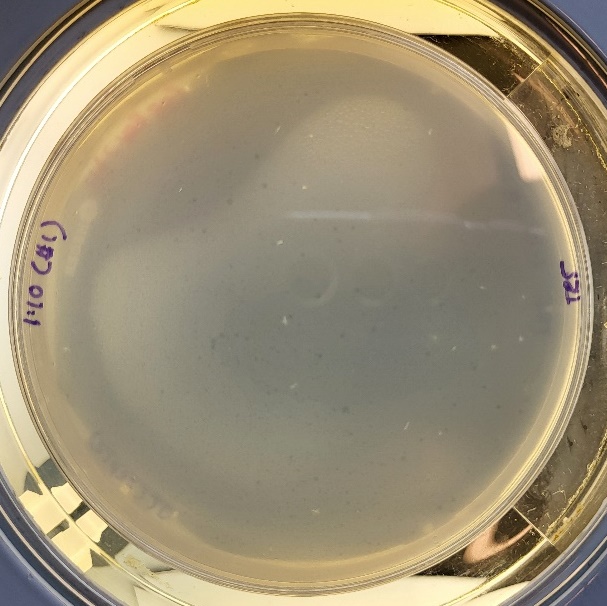

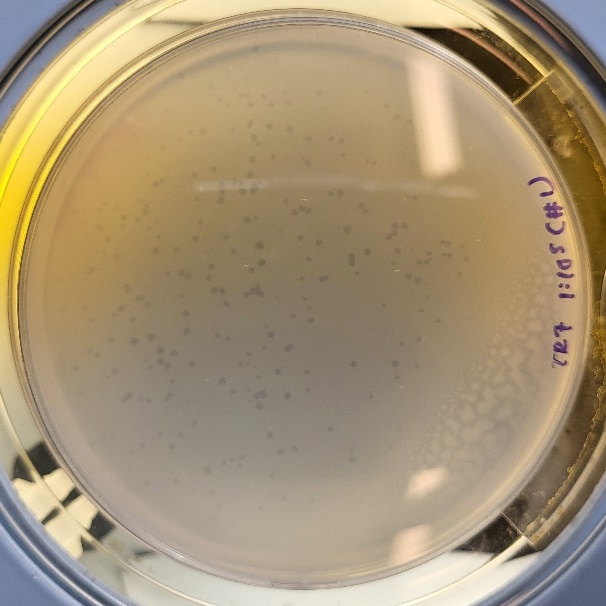

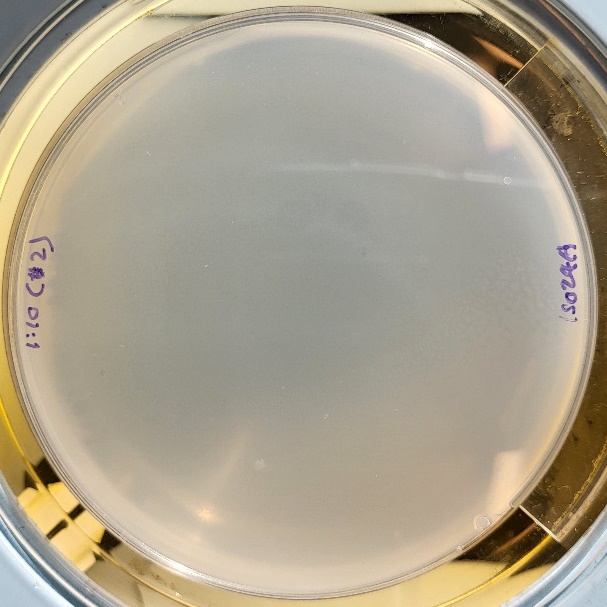


F

E

D

C

B

A

**Figure S2.** Plaques of phage G28 in the lawn of wild-type *Escherichia coli* DSM 103246 (A, B), phage G28-resistant isolates TR5 (C, D), TR7 (E), and ISO24A (F) on M9 agar after approx. 24 h incubation at 37 °C. Plaque(s) formed on TR5 and ISO24A appeared considerably smaller and fainter in comparison to those formed on WT.


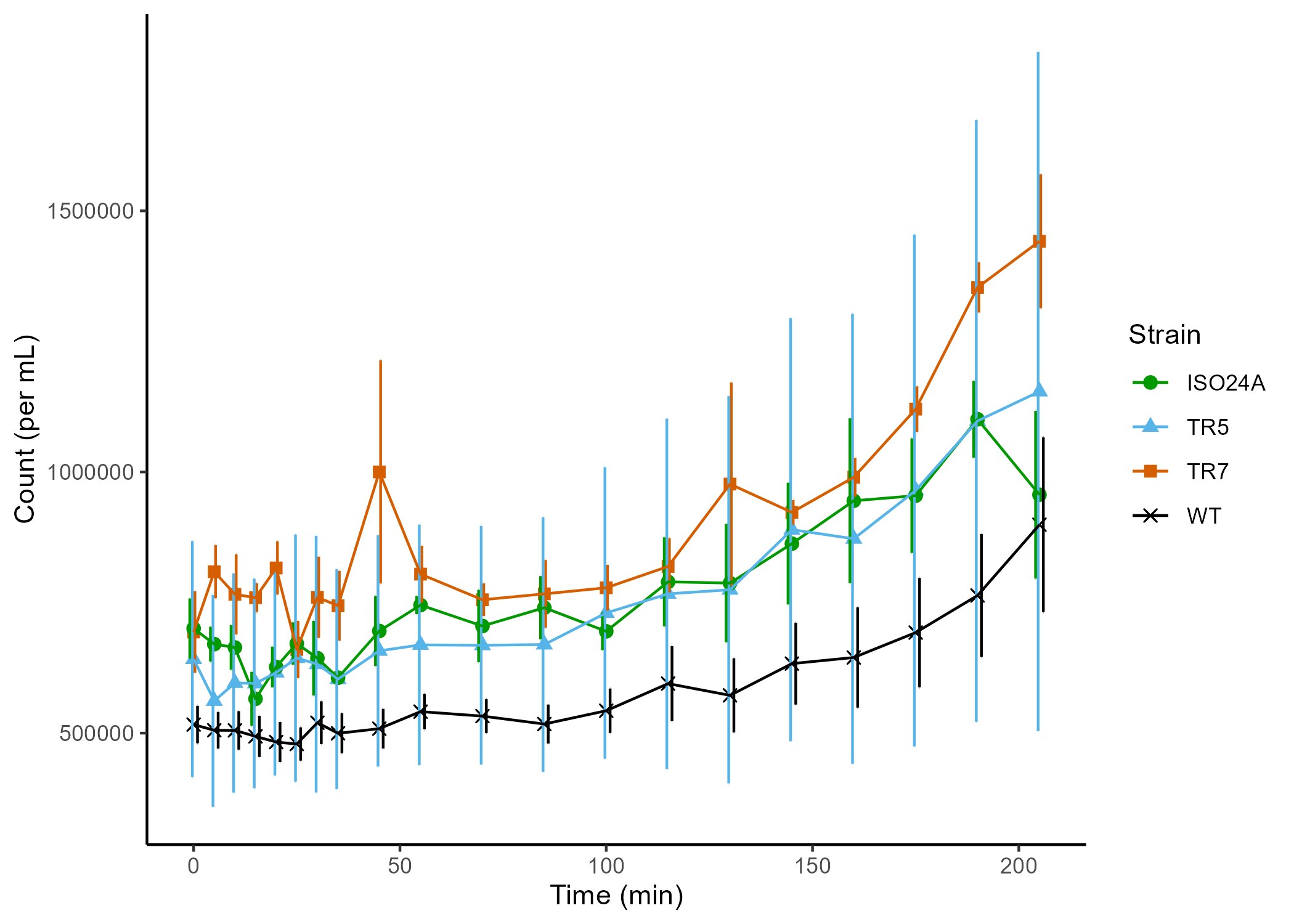


**Figure S3.** Flow cytometric count (per mL) of the wild-type *E. coli* DSM 103246 (WT) and phage G28-resistant (TR5, TR7 and ISO24A) *E. coli* cells over the duration of incubation in 50 µM L-azidohomoalanine (AHA). Datapoints = mean (n = 3; n_WT_ = 9), error bars = +/- 1 SEM.

Comment on fig. S3.: Growth was reflected in the general increase of total cell abundance during incubation for all strains. TR7 had the highest average cell abundance, but the difference was mostly not found to be significant (Tukey HSD, p > 0.05).
